# Personal Mention

**Published:** 1916-06

**Authors:** 


### Personals.

Dr. C. L. Root, of Colorado, Texas, has just returned from New Orleans, where he took special lectures and attended clinics.

Dr. W. W. Callan, of Rotan, is in Chicago taking a post-graduate course.

Dr. W. H. Pope, Jr., has just returned from Tulane University, New Orleans, where he spent several months taking a special course in X-ray science.

Dr. Ollie Shaffer has returned from Atlanta, Ga., where he has been for the past few months attending dental college.

Dr. Will K. Logsdon, who is a member of a hospital staff at Palestine, has gone to Rochester, N. Y., where he will spend some time taking a special course.

Dr. Leland C. Ellis, of Denison, and Miss Kathleen Moore, of Dallas, were recently married.

Dr. M. M. Carrick, of Dallas, will erect a public drinking fountain in the Sims Library of Waxahachie as a memorial to his mother.

Dr. E. B. Auler, a prominent physician of Elgin, is reported quite ill with smallpox.

Dr. William Thomas, of Wills Point, has been elected assistant physician at the North Texas Hospital for the Insane at Terrell, to succeed Dr. T. E. Pearson.

Dr. Adair, of Canadian, left recently for the East to take a post-graduate course.

Dr. E. C. Beaumont, of San Saba, left recently for New Orleans, where he will take a post-graduate course and recuperate from his heavy work of the past winter.

**Change of Location.**

Dr. G. L. Jackson and family of Dublin have moved to Houston.

Dr. J. R. Saunders has moved from Falfurrias to San Marcos.

Dr. J. J. Robertson and family have moved from Corpus Christi to Kingsville.

Dr. Proctor and son of Brownwood left recently for Miles, where they will make their future home.

Dr. W. A. Whiteside and family of Laneville have moved to Timpson, and will make that their future home.

Dr. E. A. Cox, of Teague, has gone to Humble, where he will equip an emergency hospital and conduct a drug store in connection, keeping up his practice of medicine as in former years.

Dr. R. M. Hargrove, of Beaumont, has been elected to fill the chair of anatomy, Medical Department of the University of Oklahoma, situated at Oklahoma City. He and Mrs. Hargrove expect to leave for their new home about August 1st.

Dr. G. L. Jakson, of Dublin, is arranging to move to Houston, where he will form a partnership with a prominent dentist and make that his home.

